# Impact of a Cloud-Based Care Coordination Platform on False Cardiac Catheterization Laboratory Activations and Unnecessary Team Mobilization: Retrospective Cohort Study

**DOI:** 10.2196/76932

**Published:** 2026-06-19

**Authors:** William Gibson, Dawoud Al Kindi, François Brouillette, Ahmed Aldajani, Omar Chaabo, Yasmine Lachance, Elie Akl, Kshitij Badal Dandona, Giuseppe Martucci, Jean-Philippe Pelletier, Nicolo Piazza, Jeremy Y Levett, Tomer Moran, Marco Spaziano

**Affiliations:** 1 Department of Cardiology, McGill University Health Centre, 1001 Decarie Blvd, Montréal, QC, H4A 0B1, Canada, 1 (514) 934-1934; 2 Department of Internal Medicine, Division of Cardiology, College of Medicine, Imam Abdulrahman Bin Faisal University, Dammam, Saudi Arabia; 3 Department of Cardiology, Faculty of Medicine, Université de Montréal, Montréal, QC, Canada; 4 Department of Cardiology, McGill University, Montréal, QC, Canada

**Keywords:** cloud-based app, ST-elevated myocardial infarction, STEMI, false activation, clinical decision support, digital health, smartphone app, acute coronary syndrome, telemedicine, mobile health, catheterization laboratory, reperfusion time, digital communication, quality improvement, resource utilization

## Abstract

**Background:**

Rapid activation of the cardiac catheterization laboratory (CCL) for ST-segment elevation myocardial infarction (STEMI) is essential to minimize time to reperfusion. However, system-wide efforts to reduce treatment delays have been accompanied by increased false activations, defined as activations that do not result in emergent coronary intervention. False activations contribute to unnecessary team mobilization (UTM), staff fatigue, workflow disruption, and inefficient resource use.

**Objective:**

This study aimed to evaluate whether the implementation of a cloud-based care coordination platform (Stenoa) was associated with reductions in false activations and UTMs at a high-volume tertiary cardiac center.

**Methods:**

In September 2021, the McGill University Health Centre implemented Stenoa, a mobile, cloud-based STEMI coordination platform enabling systematic case validation using electrocardiographic and clinical data. A retrospective cohort study was conducted, including all CCL activations between September 2020 and December 2022. Activations were grouped as preimplementation (group 0: September 2020 to September 2021) and postimplementation (group 1: September 2021 to December 2022) periods. A false activation was defined as a CCL activation followed by case cancellation before any procedure was performed. The primary outcome was the rate of UTM.

**Results:**

In total, 632 activations were analyzed (group 0: n=288; group 1: n=344). UTM decreased from 8.7% (23/265) to 4.4% (14/316) following platform implementation (*P*=.04). False activation frequency decreased from 10.2% (27/265) to 6.9% (22/316), although this difference did not reach statistical significance (*P*=.16). Among false activations, the proportion resulting in UTM declined from 85% to 63% (*P*=.08).

**Conclusions:**

The implementation of a cloud-based STEMI coordination platform was associated with a significant reduction in unnecessary catheterization laboratory team mobilization. Structured digital communication may improve workflow efficiency and resource use in STEMI systems of care. Further multicenter evaluation is warranted.

## Introduction

Every minute of ischemia counts in acute coronary syndrome (ACS), and improving time to reperfusion has become a core priority of health systems worldwide [1]. Current guidelines recommend primary percutaneous coronary intervention within 90 to 120 minutes (depending on the initial presenting location) from first medical contact (FMC) to coronary blood flow restoration as a system goal [2]. Over the past 2 decades, concerted system-level interventions have led to substantial reductions in reperfusion delay, an achievement accompanied by corresponding declines in in-hospital mortality [3,4]. These advances reflect the success of coordinated, protocol-driven pathways that emphasize early recognition, prehospital electrocardiogram (ECG) acquisition, and direct activation of the cardiac catheterization laboratory (CCL) [5]. Efficient ST-segment elevation myocardial infarction (STEMI) care depends on the seamless integration of 2 interdependent components: the STEMI procedure**,** referring to definitive revascularization via primary percutaneous coronary intervention once the patient reaches the CCL, and the STEMI process, which encompasses the upstream sequence of assessments, communications, and logistical actions that move patients from symptom onset through evaluation, triage, transport, and activation. At the center of this process lies accurate ECG-based identification of true STEMI, but fewer than 5% of chest pain presentations represent true STEMI, and while essential to STEMI diagnosis, ECG interpretation is imperfect, and several mimicking conditions can lead to unnecessary CCL activation if misidentified. As such, systems must balance rapid activation with diagnostic accuracy to deliver timely care while avoiding overtriage.

As systems have optimized speed and broadened access through earlier upstream activation pathways, a parallel concern has emerged: an increase in false or inappropriate activations, highlighting the need to achieve timeliness while maintaining diagnostic specificity. False activations—defined as CCL activations for patients who ultimately do not require emergent coronary angiography—have gained increasing recognition as an unintended by-product of system-wide efforts to shorten reperfusion times. Reported rates vary widely, from approximately 5% to 40%, reflecting heterogeneity in definitions, emergency medical service training, and activation workflows across regions [6]. Their impact is considerable: unnecessary team mobilizations (UTMs) contribute to staff fatigue and resource strain, impose avoidable financial costs [6], and may undermine patient confidence when activations are later deemed unwarranted. As health systems continue to prioritize timeliness, preserving diagnostic precision while minimizing inappropriate activations has become an important quality challenge in contemporary STEMI care.

Recently, mobile apps, telemedicine, and cloud-based platforms have markedly improved communication and coordination within the emergency care setting [7,8]. Digital transmission of prehospital ECGs to interventional cardiologists enables earlier diagnosis, triage, and direct activation of the CCL—streamlining workflow and reducing reperfusion delay. In a recent systematic review and meta-analysis of 17 studies including >4300 patients, prehospital digital ECG transmission was associated with significant reductions in door-to-device time (mean difference −33 minutes, 95% CI −50.5 to −16.2), FMC-to-device time (mean difference −24.7 minutes, 95% CI −37.1 to −12.3), and a 47% lower mortality compared with standard care (odds ratio 0.53, 95% CI 0.40‐0.69) [9]. Similarly, a recent meta-analysis of smartphone-based STEMI coordination apps demonstrated consistent time-efficiency benefits, with pooled reductions of approximately 19 minutes in door-to-balloon time and 20 minutes in FMC-to-balloon time compared with conventional activation methods, without compromising the accuracy of triage or procedural outcomes [10]. Although false activation rates could not be pooled due to variability in definitions, several included studies suggested a trend toward fewer inappropriate activations with structured app-based communication. These findings highlight the growing role of digital health tools in optimizing STEMI pathways and suggest that standardized, platform-based coordination may enhance both timeliness and accuracy of CCL activation.

Stenoa is a cloud-based mobile app dedicated to improving the CCL activation pathway by creating a single direct communication channel between the various members of the chain of survival (paramedics, emergency physicians, interventional cardiologists, and CCL teams), rather than using traditional means of communication (telephone, locating, fax, email, and SMS). Previous work from our center showed that implementation of the Stenoa platform was associated with reduced door-to-balloon times [11]. The aim of this study was to evaluate the impact of Stenoa implementation on false activation rates and the resultant effect on UTM at a single center.

## Methods

### Study Design and Setting

This retrospective observational study was conducted and reported in accordance with the STROBE (Strengthening the Reporting of Observational Studies in Epidemiology) guidelines. The completed STROBE checklist is included as Multimedia Appendix 1. This study evaluated all consecutive CCL activations at the McGill University Health Centre (MUHC) from September 2020 to December 2022, comparing outcomes before and after implementation of the mobile app. MUHC is a tertiary referral center at which personnel are off-site outside of regular working hours. Included patients were aged ≥18 years with a suspected unstable cardiac condition requiring emergent activation of the CCL. STEMI was the primary indication for CCL activation. Other indications for which the CCL was solicited included high-risk non-STEMI (NSTEMI), temporary transvenous pacemaker insertion, mechanical circulatory support for cardiogenic shock, and pericardiocentesis for cardiac tamponade. Before Stenoa implementation, CCL activation (and deactivation) was undertaken by the interventional cardiologist or emergency physician through the hospital locating services using a paging system. ECGs were either described over the phone or shared via SMS. After Stenoa implementation, CCL activation was done exclusively by the interventional cardiologist through the platform after clinical and electrocardiographic data were submitted and assessed. Similarly, prompt case deactivation could be achieved through the platform.

### Stenoa Workflow

**Figure 1.**
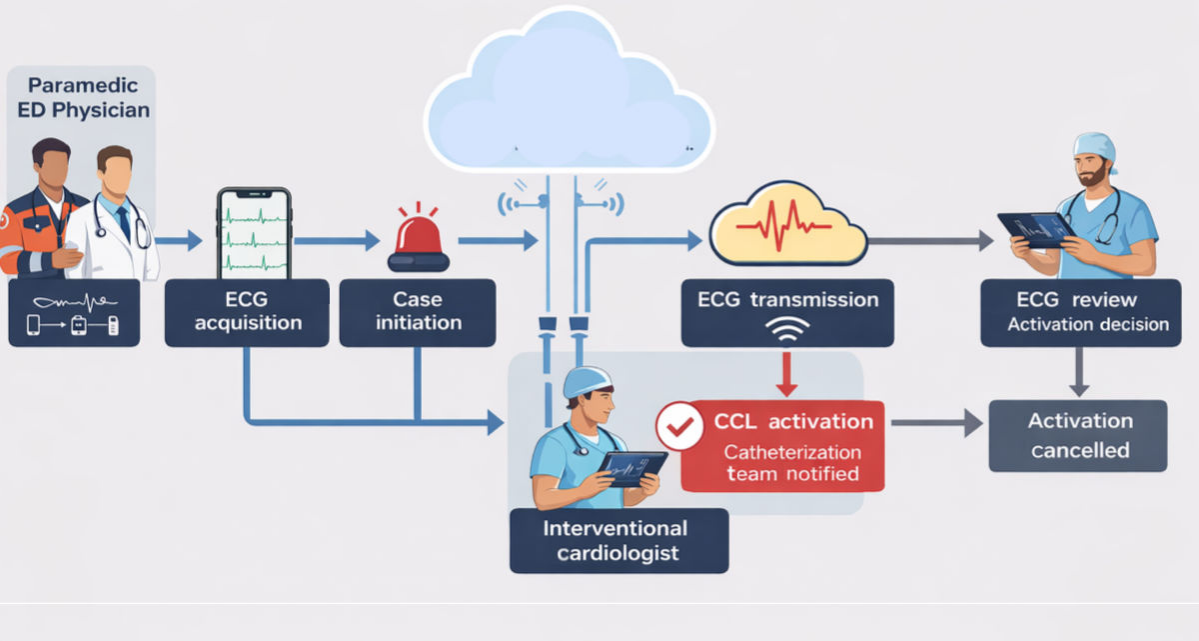
Stenoa platform workflow for cardiac catheterization laboratory activation. CCL: cardiac catheterization laboratory; ECG: electrocardiogram; ED: emergency department.

The Stenoa platform is a secure, cloud-based mobile app designed to streamline communication and decision-making during suspected ACS presentations requiring potential CCL activation (Figure 1). Patient encounters originate either from direct emergency department presentation or from the community via paramedic services. The FMC provider performs the initial clinical assessment based on symptoms, physical examination, and 12-lead ECG. When STEMI or another emergent cardiac condition warranting urgent CCL activation is suspected, a case is initiated within the Stenoa app, including patient demographics, clinical details, and ECG tracing. All uploaded information is fully encrypted and compliant with the Health Insurance Portability and Accountability Act (HIPAA) and the Systems and Organization Controls 2 security standards.

Upon case creation, coordinated real-time alerts are automatically issued to the on-call interventional cardiologist and CCL team. The interventional cardiologist reviews the submitted information through the platform and determines whether immediate activation of the CCL is appropriate. Secure messaging and integrated voice communication allow bidirectional discussion between the referring clinician and interventional cardiologist to clarify diagnostic uncertainty, if required. The final decision—activation or cancellation—is entered directly into the platform, prompting automated notification to all relevant team members (nursing staff, technicians, catheterization laboratory coordinator, and anesthesia team, if applicable).

In addition to facilitating the clinical workflow, the platform automatically captures key system performance time interval metrics, including FMC-to-ECG time, ECG-to-CCL activation time, FMC-to-balloon time, and CCL activation-to-balloon time. These time stamps are stored in real time to support internal auditing, quality assessment, and performance benchmarking.

Figure 1 presents a schematic representation of the Stenoa cloud-based communication platform used for suspected ACS requiring potential CCL activation. Following FMC, clinicians initiate a case within the platform and upload electrocardiographic data and clinical information. The on-call interventional cardiologist reviews the case in real time and determines whether CCL activation or cancellation is appropriate, with automated notifications sent to the catheterization team.

### Definitions

False activation was defined as activation of the CCL team for which no procedure was performed. UTM was defined as any false activation case that resulted in team mobilization to the hospital. False activation rates and the proportion resulting in UTM were compared before and after implementation of the app. Final diagnoses were determined based on the treating physician’s documentation in the medical record following clinical evaluation and catheterization findings. The primary outcome of this study was the overall rate of UTM.

### Participants

Patients were divided into 2 groups for analysis. Group 0 consisted of all CCL activations during the 12 months before the implementation of Stenoa (September 1, 2020, to August 31, 2021). Group 1 consisted of all CCL activations during the 15 months immediately after Stenoa’s introduction. The first 3 months (September 1, 2021, to November 30, 2021) were a transition period in which both systems were used. During the following 12 months (December 1, 2021, to December 31, 2022), CCL activations (and deactivations) were done exclusively via the platform.

Data were obtained from institutional medical records and the Stenoa platform database. Chart review was authorized by the Direction of Professional Services of the MUHC as part of a quality and outcomes evaluation.

UTM events were identified using institutional overtime billing records. These records provide an objective and auditable measure of CCL team mobilization, as clinical personnel are compensated on a per-case basis when physically mobilized to the hospital outside scheduled hours. Because CCL staff are typically mobilized from home during off-hours, overtime billing logs serve as a reliable surrogate for off-hours team mobilization events. Accordingly, the UTM metric reflects the operational burden of false activations requiring off-hours staff mobilization rather than all canceled activations occurring across the full 24-hour workflow. False activations that occurred while the CCL team was already on site (ie, during weekdays or because of a previous case) or false activations that were deactivated within 15 minutes of activation were not considered to have led to UTM. Accordingly, overtime billing logs were used as the most objective and auditable measure of team mobilization in cases of false or canceled activations.

### Ethical Considerations

This study was conducted as a retrospective observational analysis using anonymized clinical and operational data obtained from institutional medical records and the Stenoa platform. No direct patient contact or intervention occurred as part of the study. In accordance with institutional policies [12] governing retrospective analyses using deidentified data, formal research ethics board review and individual informed consent requirements were waived by the MUHC. All data were deidentified prior to analysis and handled in accordance with institutional privacy and confidentiality policies. No identifiable patient images are included in this manuscript or supplementary materials. Participants received no compensation, as the study involved retrospective analysis of existing clinical records.

### Statistical Methods

Categorical variables were summarized as frequencies and percentages, and between-group differences were evaluated using chi-square tests. Continuous variables were assessed for normality and presented as mean (SD) or median (IQR), as appropriate. A 2-sided *P* value <.05 was considered statistically significant. All analyses were conducted using standard statistical software. Data were recorded prospectively in a secure institutional database and subsequently exported for analysis.

## Results

In total, 632 patients were included in this study: 288 (45.6%) in group 0 (preimplementation group) and 344 (54.4%) in group 1 (postimplementation group). Of these, 92% (265/288 in group 0 and 316/344 in group 1) of activations occurred off-hours in both groups. The overall rate of UTM declined from 8.7% (23/265) in group 0 to 4.4% (14/316) in group 1 (*P*=.04). In total, 27 of 265 (10.2%) false activations occurred in group 0, compared with 22 of 316 (7%) false activations in group 1 (*P=*.16; Table 1). In group 0, 23 of 27 (85.2%) false activations resulted in UTM, compared with 14 of 22 (63.6%) false activations in group 1 (*P*=.08). Detailed demographic data were not available for the full study cohort; age and sex data were available only among patients with false activations. Among patients with false activations, those in group 1 were older on average (mean 71.1, SD 16.1 years) than those in group 0 (mean 67.5, SD 17.0 years), although this difference was not statistically significant (*P*=.44). In addition, the proportion of male patients was higher in group 1 (18/22, 81.8%) than that in group 0 (15/27, 55.6%), whereas female representation was greater in group 1 (15/27, 56%; *P*=.02).

**Table 1.** Rates of false activations and unnecessary team mobilization.

Outcome	Group 0^a^	Group 1^b^	*P* value
Unnecessary team mobilization, n (%)	23 (8.7)	14 (4.4)	.04
False activations, n (%)	27 (10.2)	22 (7)	.16
Unnecessary team mobilization among false activations (%)^c^	85.2	63.6	.08

a Group 0: n=265.

b Group 1: n=316.

c Percentages are calculated as the proportion of false activations that resulted in unnecessary team mobilization.

Reasons for false activations are shown in Table 2 and were similar between groups (*P*>.05). Across both cohorts, the most common cause was an alternative diagnosis other than ACS (26/49, 53.1%), including pericarditis, arrhythmias, and old myocardial infarction with persistent ST elevation. Other ACS presentations not requiring immediate angiography (NSTEMI or unstable angina) accounted for 30.6% (15/49), and factors related to patient suitability (eg, dementia, advanced comorbidity, and palliative care) accounted for 12.2% (6/49). Additionally, 1 of 49 (2%) cases was redirected to another CCL, and another involved a patient presenting with recurrent chest pain and equivocal ECG changes 4 days after an index infarction. This patient was subsequently found to have ongoing ischemia requiring repeat intervention. This event reflected a delayed presentation outside the typical primary PCI window rather than a missed activation attributable to the coordination platform. Reasons for UTM were similarly analyzed. In group 0, 39.1% (9/23) of UTM cases were unrelated to ACS, 43.5% (10/23) involved ACS presentations that did not require emergent angiography, and 17.9% (4/23) involved patients with advanced cancer receiving palliative care. In group 1, 57.1% (8/14) of UTM cases were not ACS related, 28.6% (4/14) were ACS presentations requiring delayed angiography, and 14.3% (2/14) involved prohibitive comorbidities or palliative status.

**Table 2.** Causes of false activations before and after implementation.

Cause of false activation	Group 0 (before Stenoa)^a^	Group 1 (after Stenoa)^b^
Alternative diagnosis (non-ACS^c^), n (%)	13 (48.1)	13 (59.1)
Other ACS not requiring emergent angiography, n (%)	10 (37)	5 (22.7)
Palliative care or advanced malignancy, n (%)	4 (14.8)	0 (0)
Comorbidities, n (%)	0 (0)	2 (9.1)
Late-presenting myocardial infarction or redirected case, n (%)	0 (0)	2 (9.1)

a Group 0: n=27.

b Group 1: n=22.

c ACS: acute coronary syndrome.

## Discussion

### Principal Findings

This study demonstrates a significant reduction in UTM following implementation of a cloud-based care coordination platform, decreasing from 23 (8.7%) cases before implementation to 14 (4.4%) cases after implementation (*P*=.04). Although the reduction in false activations did not reach statistical significance, the proportion of false activations that resulted in UTM decreased meaningfully from 85% to 64%, indicating more precise activation decision-making.

These improvements may be explained by the mechanisms inherent to the platform workflow. First, Stenoa increases the amount of diagnostic and contextual information available to the interventional cardiologist prior to activation, reducing reliance on incomplete early data and minimizing “blind” activations. Second, real-time secure communication enables rapid case reassessment and deactivation in response to evolving clinical information, preventing unnecessary mobilization even when activation is initially triggered. Third, structured team communication eliminates fragmented parallel notification pathways that often delay clarification or cancellation in conventional systems. Consequently, cases previously likely to progress to full mobilization despite diagnostic uncertainty may now be clarified earlier in the decision chain, lowering UTM burden without sacrificing sensitivity. Importantly, the reduction in UTM should be interpreted as an operational improvement rather than solely as a change in diagnostic accuracy. In conventional paging systems, once activation is initiated, cancellation frequently occurs after staff have already begun mobilization. By enabling earlier clarification of borderline cases, structured digital communication may prevent unnecessary mobilization before staff departure, thereby reducing operational burden even when the initial activation threshold remains unchanged. Furthermore, no cases of delayed STEMI recognition attributable to the activation workflow were identified during the study period.

The reported incidence of false activations varies widely across STEMI systems, largely due to differing definitions, diagnostic criteria, and activation workflows used in prior studies. Early prospective registry data from Larson et al [13] demonstrated a false activation rate of 14% among 1345 consecutive activations within a regional STEMI network; however, after excluding patients with ST elevation and positive biomarkers (who meet the universal STEMI definition despite the absence of an angiographic culprit), the true false activation rate was closer to 9.2%. In contrast, Youngquist et al [14] found substantially higher false activation rates with prehospital ECG–based activation than with emergency-physician activation (39% vs 9%; *P*=.02), although this was limited by small sample size and reliance on automated ECG interpretation alone. Other centers report rates ranging from 11% to 15% using coronary angiography–based definitions, such as the emergency-physician activation cohort in the study by Kontos et al [15] (14.9% false positive activations) [16] and the registry of 489 activations by Nfor et al [17] (11% without culprit lesion). McCabe et al [18] reported a higher false activation rate of 36% in the activate-SF registry using composite criteria integrating angiographic, clinical, and biomarker data; however, their definition classified patients with elevated biomarkers but without an angiographic culprit as false positives—despite technically fulfilling universal STEMI diagnostic criteria [16]—illustrating the degree of variability created by variations in the definitions adopted [18]. Similarly, statewide system data from the RACE program demonstrated inappropriate activation rates between 15% and 18.7%, depending on classification methodology [19]. Collectively, these studies highlight the absence of standardized false activation definitions and demonstrate that reported rates may differ 3-fold depending on the operational framework used, underscoring the challenge of benchmarking performance across systems. Meanwhile, the false activation rate in our center (9.4% preimplementation group) lies at the lower end of the published range. This likely reflects both definitional differences and the inclusion of high-risk NSTEMI presentations in our center, an approach that aligns with contemporary thinking around acute coronary occlusion. Growing evidence demonstrates that a substantial proportion of patients with acute coronary occlusion do not meet classic STEMI ECG criteria yet experience comparable infarct burden and benefit from urgent reperfusion. The emerging occlusive myocardial infarction model suggests that relying solely on ST-segment elevation thresholds likely risks delaying treatment among a significant cohort of patients with true coronary occlusion. In this context, many cases traditionally labeled as “inappropriate” or “false” activations may instead represent clinically justified attempts to avoid missed occlusive myocardial infarction [20]. These realities emphasize the importance of activation processes that allow clinicians to apply judgment and communicate rapidly when uncertainty exists, rather than depending solely on binary ECG criteria. Workflow solutions that enhance such collaboration—like our platform—may therefore contribute to more appropriate CCL activation.

### Limitations

#### Clinical Limitations

First, this was a single-center study, which may limit generalizability. Second, the baseline false activation rate in our institution was relatively low (approximately 10%), reducing statistical power to detect differences in false activation incidence. We applied a clinically oriented definition of false activation that did not classify cases with ECG findings warranting emergent angiography but without an occluded vessel (eg, Takotsubo cardiomyopathy) as false activations. Although some prior publications categorize such cases as false activations, doing so retrospectively risks misclassification bias, as these patients typically present with symptoms and ECG features indistinguishable from true STEMI at the point of decision-making. Third, our study does not include all chest pain evaluations in the emergency care pathway; therefore, we could not assess false-negative activations (ie, missed STEMI) in the broader population. Fourth, catheterization laboratory staff experience and satisfaction were not assessed, and future work may explore operational impact beyond mobilization frequency. Fifth, this study lacks complete demographic data for the overall cohort, as age and sex information were only available for patients with false activations. Consequently, baseline comparability between the full preimplementation and postimplementation groups could not be formally assessed. Within the false activation subgroup, differences in sex distribution and a nonsignificant difference in age were observed, suggesting potential population heterogeneity. However, given the small size of this subgroup and the system-level nature of the intervention, these differences are unlikely to fully account for the observed reduction in UTM. Nevertheless, residual confounding cannot be excluded. Finally, the study period overlapped with the COVID-19 pandemic; however, review of each false activation case did not identify instances in which pandemic-associated ECG abnormalities influenced activation decisions.

#### Operational Limitations

Implementation of the platform coincided with a change in activation workflow whereby final activation authority was centralized to the interventional cardiologist rather than shared between emergency physicians and cardiologists as in the previous system. As such, it is not possible to fully separate the effects of the digital platform itself from the impact of this workflow modification. Furthermore, detailed workflow timing metrics, such as ECG-to-activation decision time, were not available within the present dataset. However, prior work from our center evaluating the Stenoa platform demonstrated significant reductions in door-to-balloon time without delays in activation decision-making [11]. The definition of UTM was based on overtime billing records, which accurately capture episodes of team mobilization but do not account for situations in which staff were mobilized multiple times within short intervals or were delayed from leaving the hospital, potentially underestimating the practical burden on personnel and workflow. In addition, causes of false activation were determined from treating physician documentation in unblinded clinical records rather than by independent blinded adjudication, which may introduce observer bias and potential misclassification. This method of assessing UTM primarily captures off-hours mobilization burden and may not fully represent cancellations occurring during regular staffed hours. Additionally, substantial heterogeneity exists across centers in false activation definitions and activation criteria, influenced by differences in local protocols, patient populations, and operational thresholds. These challenges underscore the current need for standardized terminology and reporting frameworks to enable more meaningful comparison across systems [21]. In addition, the first 3 months following platform implementation represented a transition period during which both the traditional paging system and the Stenoa platform were used. Because individual cases could not be reliably attributed to a single system during this interval, sensitivity analyses excluding the transition period could not be performed. This may introduce a small degree of misclassification when comparing preimplementation and postimplementation periods. In this study, the consideration of UTM was grounded in resource use: if the CCL team was mobilized for a case that ultimately did not result in emergent intervention, the activation was considered unnecessary from a system perspective. Although cost-benefit analysis was beyond the scope of this manuscript, a reduction in UTMs could result in lower resource use over time.

### Conclusions

Implementation of a cloud-based STEMI coordination platform was associated with a significant reduction in unnecessary catheterization laboratory team mobilization. Therefore, digital coordination tools may represent an effective strategy for improving workflow efficiency and resource use in STEMI systems of care.

## Supplementary material

10.2196/76932 Multimedia Appendix 1 STROBE checklist.

## Abbreviations

ACS: acute coronary syndrome
CCL: cardiac catheterization laboratory
ECG: electrocardiogram
FMC: first medical contact
HIPAA: Health Insurance Portability and Accountability Act
MUHC: McGill University Health Centre
NSTEMI: non–ST-segment elevation myocardial infarction
STEMI: ST-segment elevation myocardial infarction
STROBE: Strengthening the Reporting of Observational Studies in Epidemiology
UTM: unnecessary team mobilization
