# Supplementary material for: Impact of a Cloud-Based Care Coordination Platform on False Cardiac Catheterization Laboratory Activations and Unnecessary Team Mobilization: Retrospective Cohort Study
Source: JMIR Cardio. 2026 Jun 19;10:e76932. doi: 10.2196/76932 (PMC13282013; doi:10.2196/76932)
Supplement: Multimedia Appendix 1 [file cardio-v10-e76932-s001.pdf]

STROBE Statement—checklist of items that should be included in reports of observational studies

	Item No.	Recommendation	Page No.	Relevant text from manuscript
**Title and abstract**	1	(*a*) Indicate the study's design with a commonly used term in the title or the abstract	1	"A retrospective cohort study"
		(*b*) Provide in the abstract an informative and balanced summary of what was done and what was found	1	
**Introduction**				
Background/rationale	2	Explain the scientific background and rationale for the investigation being reported	3	"Over the past two decades, concerted system-level interventions have led to substantial reductions in reperfusion delay, an achievement accompanied by corresponding declines in in-hospital mortality…"
Objectives	3	State specific objectives, including any prespecified hypotheses	5	The aim of this study was to evaluate the impact of STENOA implantation on FA rates, and any resultant effect on unnecessary team mobilisation at a single centre.
**Methods**				
Study design	4	Present key elements of study design early in the paper	5,6	This retrospective cohort study evaluated all consecutive CCL activations at the MUHC from September 2020 to December 2022, comparing outcomes before and after implementation of the mobile application
Setting	5	Describe the setting, locations, and relevant dates, including periods of recruitment, exposure, follow-up, and data collection	5,6	"
Participants	6	(*a*) *Cohort study*—Give the eligibility criteria, and the sources and methods of selection of participants.	5,6	Included patients were >18 years of

		Describe methods of follow-up		age with a suspected unstable cardiac condition which necessitated emergent activation of the CCL.
		(*b*) *Cohort study*—For matched studies, give matching criteria and number of exposed and unexposed	N/A	
Variables	7	Clearly define all outcomes, exposures, predictors, potential confounders, and effect modifiers. Give diagnostic criteria, if applicable	7	FA was defined as an activation of the CCL team for which no procedure was performed. Unnecessary team mobilization (UTM) was defined as any FA case that resulted in team mobilization to the hospital.
Data sources/ measurement	8*	For each variable of interest, give sources of data and details of methods of assessment (measurement). Describe comparability of assessment methods if there is more than one group	8	"overtime billing logs were utilized as the most objective and auditable measure of team mobilization in cases of false or cancelled activations."
Bias	9	Describe any efforts to address potential sources of bias	N/A	
Study size	10	Explain how the study size was arrived at	N/A	

Continued on next page

Quantitative variables	11	Explain how quantitative variables were handled in the analyses. If applicable, describe which groupings were chosen and why	N/A	
Statistical methods	12	(*a*) Describe all statistical methods, including those used to control for confounding	9	
		(*b*) Describe any methods used to examine subgroups and interactions	N/A	
		(*c*) Explain how missing data were addressed		
		(*d*) *Cohort study*—If applicable, explain how loss to follow-up was addressed<br>*Case-control study*—If applicable, explain how matching of cases and controls was addressed<br>*Cross-sectional study*—If applicable, describe analytical methods taking account of sampling strategy		
		(*e*) Describe any sensitivity analyses	N/A	
**Results**				
Participants	13*	(a) Report numbers of individuals at each stage of study—eg numbers potentially eligible, examined for eligibility, confirmed eligible, included in the study, completing follow-up, and analysed		
		(b) Give reasons for non-participation at each stage		
		(c) Consider use of a flow diagram		
Descriptive data	14*	(a) Give characteristics of study participants (eg demographic, clinical, social) and information on exposures and potential confounders	9	Patients in Group 1 were older on average (71.1 ± 16.1 years) compared with Group 0 (67.5 ± 17.0 years; p = 0.44). The proportion of male patients was higher in Group 0 (82%) relative to Group 1 (44%), whereas female representation was greater in Group 1 (56%; p = 0.018).
		(b) Indicate number of participants with missing data for each variable of interest		
		(c) *Cohort study*—Summarise follow-up time (eg, average and total amount)	N/A	
Outcome data	15*	*Cohort study*—Report numbers of outcome events or summary measures over time	*9*	
		Case-control study—Report numbers in each exposure category, or summary measures of exposure		
		Cross-sectional study—Report numbers of outcome events or summary measures		
Main results	16	(*a*) Give unadjusted estimates and, if applicable, confounder-adjusted estimates and their precision (eg, 95% confidence interval). Make clear which confounders were adjusted for and why they were included	9	
		(*b*) Report category boundaries when continuous variables were categorized		
		(*c*) If relevant, consider translating estimates of relative risk into absolute risk for a meaningful time period		

Continued on next page

Other analyses	17	Report other analyses done—eg analyses of subgroups and interactions, and sensitivity analyses	N/A	
**Discussion**				
Key results	18	Summarise key results with reference to study objectives	11	This study demonstrates a significant reduction in unnecessary team mobilization (UTM) following implementation of a cloud-based care coordination platform, decreasing from 23 cases (8.0%) before implementation to 14 cases (4.1%) after implementation (p = 0.04).
Limitations	19	Discuss limitations of the study, taking into account sources of potential bias or imprecision. Discuss both direction and magnitude of any potential bias	13	
Interpretation	20	Give a cautious overall interpretation of results considering objectives, limitations, multiplicity of analyses, results from similar studies, and other relevant evidence	12	The reported incidence of false activations varies widely across STEMI systems, largely due to differing definitions, diagnostic criteria and activation workflows used in prior studies.
Generalisability	21	Discuss the generalisability (external validity) of the study results	14	"Although some prior publications categorize such cases as false activations, doing so retrospectively risks misclassification bias, as these patients typically present with symptoms and ECG features indistinguishable from true STEMI at the point of decision-making."
**Other information**				
Funding	22	Give the source of funding and the role of the funders for the present study and, if applicable, for the original study on which the present article is based	20	

*Give information separately for cases and controls in case-control studies and, if applicable, for exposed and unexposed groups in cohort and cross-sectional studies.

Note: An Explanation and Elaboration article discusses each checklist item and gives methodological background and published examples of transparent reporting. The STROBE checklist is best used in conjunction with this article (freely available on the Web sites of PLoS Medicine at http://www.plosmedicine.org/, Annals of Internal Medicine at http://www.annals.org/, and Epidemiology at http://www.epidem.com/). Information on the STROBE Initiative is available at www.strobe-statement.org.
